# Development and validation of Chinese college students’ future employability scale

**DOI:** 10.3389/fpsyg.2023.1063437

**Published:** 2023-02-23

**Authors:** Wanyu Chen, Kaixu Shao, Qiuye Xiao, Yilan Mai

**Affiliations:** ^1^Department of Psychology, Faculty of Education, Guangxi Normal University, Guilin, China; ^2^Guangxi University and College Key Laboratory of Cognitive Neuroscience and Applied Psychology, Guilin, China

**Keywords:** college students, future employability, reliability, validity, scale development

## Abstract

COVID-19 and the pandemic-induced lockdowns juxtaposed against the surge in the number of college graduates have made the dilemma of “fierce competition and difficult employment” more real. The employment of college students has become a topic of serious concern in society. This study aimed to develop a Future Employability Scale for Chinese college students and evaluate its reliability and validity. Based on the analysis of the literature, the study developed the initial measurement scale of the college students’ future employability and calibrated the initial measurement and question volume based on experts’ feedback. First, the students’ group was measured, and data from 389 university students were collected and analyzed. Second, the data collection and verification factor analysis of 387 university students were collected and verified, and the internal consistency reliability, split-half reliability, and validity of the scale were evaluated. Further, 68 college students were selected to evaluate their test-retest reliability after an interval of one month. The Future Employability Scale of college students had 28 items covering four dimensions: knowledge skill, personality quality, interpersonal network, and career development. The reliability test found that the total scale of the Future Employability Scale and the internal consistency reliability, split-half reliability, and retest reliability of each dimension were good, and the validity test suggested that the scale had good content validity, structural validity, and calibration correlation validity. With a clear structure, good reliability, and validity, the Future Employability Scale is a good tool to measure the future employability of college students.

## Introduction

In the 21st century of information integration and economic globalization, individual career development is characterized by fluidity and uncertainty. The era of borderless careers emphasizes the promotion of employability instead of long-term employment guarantees so that individuals can get opportunities to continue to work across different organizations. According to recent statistics, the number of college graduates in China reached a record high of 10.76 million in 2022 (Chen and Wang, 2022). This increase in the number of graduates at a time when COVID-19 is spreading (and impacting all sectors), making the dilemma of “fierce competition and difficult employment” very real. It has also resulted in higher requirements for the development of college students’ employability. Against this background, the General Office of the Ministry of Education has issued several Employment Guidance Policy Outlines such as the “14th Five-year Plan” Employment Promotion Plan aimed at improving the employability of college students through training and helping them make better choices in future career development (Liu et al., 2021).

In the face of uncertainty, constructing forward-looking self-representation could help to predict future events, set goals, make commitments, and promote future development. Future employability, as an important part of self-reflection on future work (Pisarik and Shoffner, 2009), is an individual’s expected assessment of future self-development and a portrayal of the individual’s career development at a certain point in the future (Cross and Markus, 1991; Ellen et al., 2012). According to Super’s (1980) life-span and life-space approach to the career development of young students, the career exploration period (15-24 years old) plays the most important role in the whole life stage connecting the past and the future. It is the period in which their judgment of their possible future selves drives their current career ideals, emotions, and behaviors. For college students, it is necessary to develop and improve their employability if they want to have a bright career development prospect in the era of borderless careers (Huang et al., 2022) where there is an increasing emphasis on employment across organizations as an alternative to permanent employment. In this context, to improve career competitiveness, it is not enough only to focus on the individual’s current employability but also to make a clear judgment on the possible future self-development. For college students who are about to finish their studies but are not actively preparing for their future career development, their judgment of their possible self is crucial to their future career development. As an important part of the future work self, future employability drives the individual’s current behavior and is affected by the individual’s current situation. Therefore, researching future employability can promote the improvement of college students’ current employability.

The basis of researching the future employability of college students is to clearly define the concept and provide suitable measurement tools. Given that the concept and connotation of future employability are still developing, and the measurement tools for future employability are also still developing, when seeking to understand the concept of future employability and compiling related scales, this study will also focus on understanding the concept of employability. The basic concepts and structures draw on employability measurement tools. In previous studies, different scholars have offered a different understanding of the term “employability,” which affects the academic definition of employability and future employability. Some researchers interpret the term “employability” to mean having the ability to work (Hobfoll et al., 2003; Yu et al., 2014), while others understand “employability” as perceived employability (Buchmann, 2002; Li, 2006; Hu and Zhong, 2015), thus resulting in different definitions of employability in current academic circles and different research perspectives.

With the increasing number of college graduates, their employment has been an issue of concern among various stakeholders. Some scholars have had a preliminary discussion on the concept of future employability (Gunawan et al., 2018, 2020), but it has not been systematically defined in the academic circle at present, and the concept of employability and future employability needs to be explored and clarified. Currently, the concept of future employability is not clear and the research tools are not perfect.

According to Presti et al. (2022b), it is important for individuals to successfully enter the labor market and improve their career competitiveness if they want to complete their academic tasks. In the face of uncertainty, it is also of great significance to pay attention to college students’ future employability from the perspective of career development. First, future employability is an important representation of the future possible self. College students’ current individual conditions affect their assessment of future employability, and their judgment of their future employability affects their emotions and behaviors. Second, the development of a future employability scale can provide a suitable tool for college students to evaluate their future employability and explore their future possibilities. Third, the development of a future employability scale can help colleges and universities to carry out employment guidance and career counseling more effectively.

To solve the above problems and further the research in this field, the definition of employability is first addressed in this study. Second, based on the theory of conservation of resources (Hobfoll et al., 2003), the possible selves theory (Cross and Markus, 1991), and the social positioning theory (Tony, 2022), this study examines the development process of college students’ employability, defines the concept and connotation of future employability combined with relevant research on future employability, and unpacks the structure of future employability to lay a theoretical foundation for the preparation of a scale of future employability for college students. Third, the dimensions of the scale are determined by item analysis and exploratory factor analysis. Through exploratory factor analysis, the five dimensions of the research hypothesis are reduced to four dimensions by combining the two dimensions of “job search strategy” and “career development”. In the confirmatory factor analysis, a section of the four-factor model is determined as the best model through model comparison. The Future Employability Scale of college students has a good split-half reliability, retest reliability (interval of one month), and calibration correlation validity. In the discussion section, the measurement perspective, structure, reliability, and validity of the scale of college students’ future employability, as well as the research significance, research limitations, and future research directions, are analyzed and discussed. Finally, this study proposes a Future Employability Scale that has 28 items and consists of four dimensions: knowledge and skills, personality quality, interpersonal network, and career development. The scale of future employability of college students has good internal consistency reliability, split-half reliability, retest reliability, content validity, and structure validity, which can be used as an effective tool to evaluate the future employability of college students. It also provides a basis for follow-up and further research on the future employability of college students.

### Employability

Different scholars have different understandings of the term “employability”. Some researchers, based on the conservation of resources (COR) theory, believe that employability is a kind of individual asset possessed by individuals in the workplace (Hobfoll et al., 2003; Yu et al., 2014; Jabeen et al., 2022). It provides the possibility of individual employment; Vanhercke et al. (2014) suggest that employability is an individual’s ability to obtain and maintain employment. Although there are some disputes on the definition of employability, these researchers all discuss the ability of individuals to find and maintain jobs from the perspective of employability itself. However, from the perspective of organizational behavior, other researchers believe that employability focuses on the possibility of underemployment that individuals perceive subjectively. (Buchmann, 2002; Li, 2006; Rothwell and Arnold, 2007). Some researchers have proposed that, although individual employability is based on a series of personal attributes such as knowledge and skills and learning ability, it is ultimately subject to organizational and external labor market conditions (Rothwell and Arnold, 2007). Rothwell and Arnold (2007) and Hu and Zhong (2015) distinguish employability from internal employability and external employability through two dimensions: employability as the possibility of being hired refers to an individual’s control over his or her career development based on career experience, internal employability refers to the judgment of current self-employability, and external employability refers to the judgment of the labor market (Hu and Zhong, 2015). Although employment-related abilities and the possibility of being hired are derived from the word “employability”, there is a fundamental difference between them. The possibility of being hired covers employment-related abilities to a certain extent. Employment-related abilities are equivalent to internal employability as defined by Rothwell and Arnold (2007), who focus on individual employment-related abilities.

Since the 1990s, employability has come to mean how individuals might maintain their job readiness, especially when they are at risk of having to change jobs (Guilbert et al., 2016). Holmes (2013) distinguishes between three competing explanations of university graduate employability: possession (of human capital); position (based on social capital); and process (based on career self-management (CSM)). Most researchers who have studied employability during the transition from college to work have done so from the first two perspectives (Mavromaras et al., 2010; Kalfa and Taksa, 2015). In addition, Holmes pointed out that it is necessary to study the employability of college students based on career self-management. Okay Somerville and Scholarios (2017) also pointed out that, in turbulent economic times, it is necessary to actively pay attention to college students’ employability from the perspective of career self-management. In recent times, with the spread of COVID-19 and the global economic recession, individuals are facing more and more uncertainties in their careers (James et al., 2021). Therefore, it is not enough to only focus on individual employability in terms of possession (of human capital) and position (based on social capital). It is of utmost significance to focus on the employability of individuals from the perspective of career self-management for their career development and employment guidance.

Rothwell et al. (2009) identified three perspectives from which research on employability can be undertaken: the level of the national workforce (e.g., government policies), the level of human resource management, and the level of the individual (e.g., individuals’ beliefs about their employment). However, most researchers focus on employability from the individual perspective, because individuals’ perception and judgment of their employability affect their career choice and career development. This study is also based on the individual level of current and future employability.

This study suggests that employability as an employment-related ability is a positive individual resource, which is the ability of individuals to obtain employment, maintain employment, secure re-employment and promote future career development (promotion) in the current stage. As a concept developed from the field of management, employability as the possibility of being hired is an individual’s subjective judgment of whether he or she is employable based on his or her own ability and the demand of the labor market (Hobfoll et al., 2003; McQuaid and Lindsay, 2005; Van der Heijde and Van der Heijden, 2006; Holmes, 2013; Yu et al., 2014; Song, 2017).

### Future employability

Some researchers point out that future employability focuses on an individual’s current judgment and perception of their own employability (Gunawan et al., 2018, 2020), and future employability is a self-portrait of an individual’s career development at a certain point in the future (Cross and Markus, 1991; Ellen et al., 2012). Career development self-portrait ideas about employability from the perspective of the future self are based on employability and the judgment of future working conditions at the current stage (Gunawan et al., 2018, 2020). Gunawan et al. (2020) suggests that proactive personality, career strategies, career encouragement, and career calling affect future employability, which will affect career planning, performance, and career satisfaction.

There are differences between future employability and employability (Table 1). These specific differences are reflected in the following three aspects. First, the difference in essence: employability is a kind of ability resource (Hobfoll et al., 2003; Yu et al., 2014) and future employability is a kind of self-efficacy (Gunawan et al., 2018, 2020). The possible selves can be defined as future self-representations (Lee, 2022). Future employability is not an objective ability capital, but the perception and judgment of the possible future selves based on the current situation. Second, the difference in time nodes: employability is concerned with the current status of an individual (Song, 2017) and future employability is the judgment of expectations (Cross and Markus, 1991; Ellen et al., 2012; Gunawan et al., 2018, 2020). Markus and Nurius (1986) coined the phrase possible selves to describe how an individual’s representation of self is constructed from past selves with a look toward the development of future selves (Bowen, 2016). Third, the difference in self-concept: employability relates to the actual self, and future employability relates to the possible self (Higgins, 1987; Hobfoll et al., 2003). Future employability is regarded as a concept of future orientation which is an individual’s judgment of his or her occupational self after finishing school and stepping into the workplace (Gunawan et al., 2018).

**TABLE 1:** Difference between employability and future employability.

	Employability	Future employability
Essence	Capability resource	Self-efficacy
Time- conceptually	Current situation	Looking forward to the future
Self-conceptually	Actual self	Possible selves

To sum up, we define future employability as the state of an individual’s ability in future employment, which, based on the current stage, the individual predicts and evaluates the employability he or she will have in the future from a self-perspective. Based on the current stage of employability, the individuals predict and evaluate their own employment, preserve employment, secure re-employment, and promote career development (promotion) in the future. It is a picture of the person’s career development at some point in the future (Cross and Markus, 1991; Ellen et al., 2012; Gunawan et al., 2018, 2020). In the context of our study, future employability refers to the future employability of college students, and their ability to predict and evaluate future employability based on the current stage.

College students will soon face their first employment (Wang, 2015). Therefore, we define the future employability of college students as the state of college students ‘ ability in employment based on the current stage, the individual predicting and evaluating the employability that an individual will have in the future from self’s perspective. It is said that, based on the current stage of employability, the individual predicts and evaluates their own employment, preserving employment, securing re-employment, and promoting future career development (promotion) in the future. It is a picture of the person’s career development at some point in the future.

### Measurement of future employability and the issues addressed in this study

Most of the previous studies are focused on the development of employability measurement tools, and research tools on future employability are still relatively lacking. In earlier studies, there are four main perspectives on the research on the structure of employability. The first, the static perspective, considers employability to include a range of knowledge, skills, and characteristics. This perspective focuses on the measurement of basic capabilities and professional capabilities (Song, 2008). Second, from a dynamic perspective, Hillage and Pollard (1998) put forward that employability includes the stock of capital such as knowledge, skills, and attitudes and the use of capital. Third, from the perspective of coping with career changes, Fugate et al. (2004) suggest that employability is a holistic approach formed by the interaction of career identity, personal adaptability, social capital, and human capital. Fourth, an integrated perspective based on the conservation of resources (COR) theory, Yu et al. (2010, 2014) hold that the structure of college students’ employability should be discussed from the perspective of integration.

There are two perspectives on the measurement of employability: the input-based approach and the output-based approach (De Cuyper et al., 2012). Both these perspectives regard employability as a personal resource that individuals need to obtain and maintain the possibility of employment, but they have different approaches. The input perspective emphasizes the factors that increase the possibility of employment, which is regarded as the competency characteristics of individuals to improve and maintain employment (Van der Heijde and Van der Heijden, 2006), personality characteristics (Fugate and Kinicki, 2008), and social capital characteristics (Leana and Van Buren, 1999). On the other hand, the output perspective emphasizes more on employment outcomes. It emphasizes the individual’s overall perception of the possibility of obtaining and maintaining employment (De Cuyper et al., 2011).

A positive image of an individual’s future employability should be associated with the person engaging in detailed relevant thoughts about their current status (Lord et al., 2010; Gunawan et al., 2018) and lead them to implement strategies and behaviors (i.e., self-regulate) that will maintain their progress and improve their chances of fulfilling their future image (i.e., meet their employment goals) (Gunawan et al., 2018).

In the study on future employability, Gunawan et al. (2018) highlight that employability, or the measurement of future employability, emphasizes more on employability related to obtaining jobs, maintaining employment security, and promoting career development. They also compiled a Perceived Future Employability Scale and pointed out that proactive personality, career strategies, career encouragement, and career calling affect future employability, which will affect career planning, performance, and career satisfaction (Gunawan et al., 2020). They suggest six dimensions in their perceived future employability scale including perception skills, accumulated experience, personal characteristics, interpersonal network, labor market knowledge, and graduating institution’s reputation. Gunawan et al. place more emphasis on employment results from an output-based approach. In other words, it emphasizes the overall perception of an individual on the possibility of obtaining and maintaining employment in the future (De Cuyper et al., 2011), which is an individual’s self-evaluation on whether he or she can obtain and maintain employment in the future. However, it includes labor market knowledge, educational institutions’ reputation, and other factors that affect future employability.

According to the conservation of resources (COR) theory, resource loss is the primary operating mechanism driving stress reactions. The COR theory further suggests that, faced with adversity, people mobilize remaining resources (i.e., those not lost in stress’s onset) to offset the ongoing challenges that confront them to the extent that they will limit resource loss, which will manifest fewer negative outcomes because these resources are integral to the individuals’ ability to offset stress, improve their conditions, and deter future stressful experiences (Hobfoll et al., 2003). College students’ assessment of their future employability is based on their current employability and the development of their employability. The resources they have will be indispensable for coping with future life events.

Markus and Nurius (1986) proposed the concept of the possible self. They refer to the future-based visual, semantic, or symbolic representation as the possible self. The possible self is the future component of self-consciousness, which is related to individual potential and future. The assessment of college students of their future employment is exactly the judgment of their possible future self.

Meanwhile, according to social positioning theory, more or less any item can be allocated to any community position where relevant community participants are prepared to accept or go along with it. This is, even if, for whatever reason, the powers associated with an instance are possessed, unexercised, or unfilled, and the associated function exists unrealized. However, the social positioning of an individual will give rise to a human component instance that is capable of participating or having an impact in the relevant community only where the relevant individual possesses some of the basic position-oriented capacities. Therefore, whether or not a particular component instance can perform its associated function, and indeed, in the case of a human component, whether he or she is willing to do so, will depend in some part on the nature of the particular position occupant. (Tony, 2022). Identity is a major component of social positioning theory and the judgment of identity is dynamic and changing. Therefore, college students need to look at themselves and the social environment from the perspective of development and change.

This study, therefore, starts from the concept of future employability and does not consider many factors that affect future employability such as the reputation of educational institutions. This study is based on the conservation of resources (COR) theory (Hobfoll et al., 2003), the possible selves theory (Markus and Nurius, 1986; Cross and Markus, 1991), and the social positioning theory (Tony, 2022). From the perspective of career self-management and the measurement perspective of the output-based approach, it pays more attention to skills and attributes (Holmes, 2013) and processes and pays more attention to the employment results of college students in the future. It emphasizes the overall perception and judgment of individuals on the possibility of obtaining and maintaining employment in the future to construct the dimension of future employability and develop the Future Employability Scale.

The development of the scale has a standardized process. This study followed a rigorous scale compilation process (Dai, 2015; Wang and Fu, 2018; Xiong and Ye, 2020). First, based on the relevant literature review, the initial questionnaire was written in combination with students’ actual situation and relevant tools. Second, experts and teams evaluated the scientific and operational aspects of the scale multiple times. Third, three methods were used to analyze the questions, including the high-low grouping method, the correlation degree method between the questions and the total score, and Cronbach’s α coefficient method after the deletion of the questions. Subsequently, after several exploratory factor analyses, it was summarized that the scale of future employability would include four dimensions. Confirmatory factor analysis also confirmed the structure of the first-order four factors, indicating the reliability and scientific nature of the scale. Finally, the reliability analysis of the Future Employability Scale for college students showed that the scale has good internal consistency reliability, split-half reliability, and retest reliability.

## Study 1

### The present study

The paper compiled the preliminary measurement scale of college students’ future employability and formed the preliminary questionnaire based on expert evaluation, item analysis, and exploratory factor analysis on college students.

### Methods

#### Participants

Referring to the commonly used method (Curran, 2016; Xu and Li, 2021), 11 participants whose answers were incomplete, and 389 valid participants remained in the end. Their average age was 20.24 ± 1.95; there were 130 male students (33.4%) and 259 female students (66.6%). Among them, 166 (42.7%) came from rural areas and 223 (57.3%) came from urban areas. There were 120 freshmen (31.0%), 72 sophomores (18.5%), 125 juniors (32.0%), and 72 seniors (18.5%). Of the study population, 120 (31.0%) were the only child, 269 (69%) were not the only child, 264 (68.0%) were first-generation college students, and 125 (32.0%) were non-first-generation college students in their families.

#### Process

The first step was to determine the dimensions of the future employability scale according to the literature review and preliminary investigation, to frame the questions by referring to the existing employability scale, and to finally draft 75 questions. The second step was to prepare the topic, modify its language, and express it concisely (Zhou et al., 2013). The questions with similar meanings were deleted and modified, and finally, 50 questions were decided. The third step was expert validity and evaluation. Among them, five were university teachers whose research interests were related to career assessment and human resource assessment. To better understand the requirements of the labor market for college students and investigate their future employability from multiple perspectives, this study also invited two enterprise workers to evaluate the contents of this scale, one of whom was a senior economist and the other, a founder of an enterprise. They were invited to score the questions on a scale of 1-3 based on whether they reflected future employability, where “1” indicated inappropriate, “2” indicated appropriate after modification, and “3” indicated appropriate. Finally, a total of 50 questions for the “College students future employability questionnaire” were formed. The fourth step was to determine the scoring method of the scale. The initial scale consisted of 50 questions, including the 5 dimensions of knowledge and skills, personality quality, interpersonal network, job hunting strategy, and career development, with 10 questions in each dimension. The scale adopted a five-point Likert scale on a scale of 1-5 from “ completely disagree ” to “ completely agree ”. The higher the cumulative score, the stronger the employability. Finally, item analysis and exploratory factor analysis were performed using SPSS 25.0.

## Results

### Item generation and analysis

First, the 50-item questionnaire on college students’ future employability was analyzed. The objective of the item analysis was to test the differentiation degree of the questions, which could be used as an index to exclude items of poor quality. In this study, we used the high-low grouping method, the total correlation method, Cronbach’s α coefficient method after item deletion, and the common degree method for analysis (Dai, 2015). The independent sample T-test was used to test the high and low groups that were grouped according to 27% before and after. As shown in Table 2, there were significant differences between the 50 questions in the high and low groups (*P < 0.01*), and the T-test was all greater than 3. Therefore, no question was deleted based on this method. According to the correlation analysis results between questions and the total score in Table 2, the correlation coefficients between each question and the total score ranged from 0.676 to 0.817, and no question had a correlation coefficient lower than 0.4. Therefore, the questions were not deleted based on the total correlation method. In addition, the preliminary scale of the 50 questions was judged based on whether Cronbach’s α coefficient after the item was deleted would improve the overall Cronbach’s α. The questions were not deleted in this step. The common factor method uses the common degree to judge whether the overall item is suitable for measuring the same construct. According to the results in Table 2, the common degree was between 0.592 and 0.754, because the topic was not deleted based on this method. Based on the results of the above four methods, it was established that the 50-item preliminary scale of future employability that was developed was based on a strict and standardized compilation process had a high degree of differentiation, which was suitable for measuring future employability of college students.

**TABLE 2:** Item analysis.

Item	Mean (M)	Standard deviation (SD)	Identification index (T)	Correlation between each item and total (r)	Delete Cronbach’s α after this item
FES1	3.84	0.923	23.438***	0.714**	0.985
FES 2	3.80	0.958	25.064***	0.724**	0.985
FES 3	3.72	0.967	23.25***	0.713**	0.985
FES 4	3.81	0.942	26.488***	0.775**	0.985
FES 5	3.96	0.875	28.481**	0.770**	0.985
FES 6	3.75	0.961	28.686***	0.761**	0.985
FES 7	3.75	0.966	27.305***	0.761**	0.985
FES 8	3.56	1.052	27.145***	0.758**	0.985
FES 9	3.80	0.958	26.615***	0.776**	0.985
FES 10	3.75	0.941	26.942***	0.782**	0.985
FES 11	3.75	0.921	31.134***	0.817**	0.985
FES 12	3.78	0.915	25.56***	0.773**	0.985
FES 13	3.89	0.946	24.007***	0.730**	0.985
FES 14	3.94	0.901	25.268***	0.759**	0.985
FES 15	3.89	0.931	27.512***	0.776**	0.985
FES 16	4.12	0.857	20,866***	0.684**	0.985
FES 17	4.20	0.836	21.429***	0.676**	0.985
FES 18	3.96	0.879	24.40***	0.751**	0.985
FES 19	3.95	0.880	31.23***	0.790**	0.985
FES 20	4.06	0.868	26.654***	0.760**	0.985
FES 21	3.69	0.972	28.996***	0.783**	0.985
FES 22	3.63	0.955	27.294***	0.765**	0.985
FES 23	3.75	0.946	27.706***	0.762**	0.985
FES 24	3.71	0.969	28.976***	0.765**	0.985
FES 25	3.92	0.909	30.441***	0.785**	0.985
FES 26	3.59	0.980	26.614***	0.765**	0.985
FES 27	3.99	0.873	26.308***	0.748**	0.985
FES 28	3.97	0.863	27.467***	0.778**	0.985
FES 29	3.56	1.037	23.258***	0.700**	0.985
FES 30	3.73	0.909	27.59***	0.786**	0.985
FES 31	3.90	0.887	28.938***	0.778**	0.985
FES 32	3.80	0.864	27.153***	0.780**	0.985
FES 33	3.86	0.905	30.979***	0.783**	0.985
FES 34	3.80	0.922	29.537***	0.793**	0.985
FES 35	3.78	0.923	31.31***	0.803**	0.985
FES 36	3.74	0.951	28.87***	0.794**	0.985
FES 37	3.98	0.875	30.219***	0.761**	0.985
FES 38	3.92	0.893	30.943***	0.777**	0.985
FES 39	3.84	0.924	28.484***	0.780**	0.985
FES 40	3.80	0.925	29.186***	0.777**	0.985
FES 41	3.90	0.851	27.099***	0.763**	0.985
FES 42	3.90	0.880	26.873***	0.757**	0.985
FES 43	3.76	0.884	22.784***	0.697**	0.985
FES 44	3.94	0.851	29.887***	0.782**	0.985
FES 45	3.98	0.846	29.461***	0.782**	0.985
FES 46	3.87	0.920	32.092***	0.789**	0.985
FES 47	3.81	0.924	33.507***	0.779**	0.985
FES 48	3.61	1.020	31.507***	0.763**	0.985
FES 49	3.77	0.953	29.507***	0.807**	0.985
FES 50	3.71	0.958	27.507***	0.763**	0.985

**P < 0.01 and ***P < 0.001.

### Exploratory factor analysis

Principal component analysis was used to extract common factors from the data of sample 1 (*N* = 389), and the skew rotation method was used in reference to relevant research methods (Zhou et al., 2013; Xu and Li, 2021) to perform factor rotation. Based on whether the characteristic root is greater than 1 and the problem factor load is greater than 0.63 (Comrey and Lee, 2013), no cross load was found.

## Results

The KMO value was 0.976, and the Bartlett Sphericity test (χ^2^ = 27069.366, DF = 2278; *P <* 0.001) was suitable for factor analysis. The common degree of each topic and the factor load after rotation are shown in Table 3. Four factors with characteristic roots greater than 1 were extracted from the 28 questions through principal component factor analysis, and the cumulative variance contribution rate of the four factors was 68.666%.

**TABLE 3:** Load matrix of each factor after rotation.

Item	Knowledge and skills	Personality quality	Interpersonal network	Career development	A coefficient after deletion of items
FES1	0.957				0.755
FES 2	0.857				0.733
FES 3	0.774				0.694
FES 4	0.874				0.766
FES 6	0.675				0.684
FES 7	0.678				0.673
FES 8	0.657				0.667
FES 13		0.698			0.661
FES 14		0.792			0.704
FES 16		1.019			0.730
FES 17		1.020			0.727
FES 18		0.720			0.650
FES 19		0.657			0.714
FES 20		0.741			0.670
FES 21			0.677		0.692
FES 22			0.890		0.744
FES 23			0.829		0.729
FES 24			0.841		0.716
FES 25			0.641		0.715
FES 26			0.795		0.721
FES 29			0.890		0.667
FES 38				0.641	0.665
FES 45				0.668	0.704
FES 46				0.744	0.728
FES 47			;	0.814	0.700
FES 48				0.936	0.734
FES 49				0.942	0.788
FES 50				0.908	0.703
Eigenvalue	29.096	2.316	1.620	1.300	
Variance contribution rate (%)	58.192	4.633	3.240	2.601	
Cumulative variance contribution rate (%)	58.192	62.825	66.065	68.666	

According to the screening rules of factor analysis, the item was analyzed and screened. The research followed the following principles when selecting the topics (Comrey and Lee, 2013; Wang, 2014).

(1) The item must be greater than 0.63 in its corresponding factor load.
(2) There should be no cross-loadings between each factor.
(3) The items on each factor must be greater than or equal to three and, otherwise, delete the factor.
(4) The content of the items must be relatively appropriate, and the items with improper attribution should be deleted.

The exploratory factor analysis found that the factor extraction number that we originally constructed in terms of the five dimensions of “skills”, “personality quality”, “interpersonal network”, “job search strategy”, and “professional development” were not consistent; in exploratory factor analysis, the dimensions of “job search strategy” and “career development” were ascribed together. This study accepted the four-factor model. Job search is a process for college students to complete their career transition and enter the workplace smoothly. It mainly reflects the ability of an individual to get a job. In this study, we assumed that the ability to obtain and select jobs (job search strategy) should be a separate dimension, whereas the exploratory factor analysis combined job search strategy and future career development ability together. It also proved that individual career development was continuous even in stages (Super, 1980).

The four extracted factors were described and named as follows: The first factor consisted of seven questions, which were 1, 2, 3, 4, 6, 7, and 8. These items related to the knowledge and skills that college students should possess in the future job search and working process and were named “knowledge and skills”. In the section on knowledge and skills, we deleted three questions (e.g., I will have the learning ability required for work; I will have the communication skills required by the job; I will be able to apply what I have learned to my work flexibly). The second factor had seven questions, which were 13, 14, 16, 17, 18, 19, and 20. These items related to the basic qualities that college students should possess in their daily work in the future, and were named “personality quality”. In the section on personality quality, we deleted three questions (e.g., I will have the ability to deal with work affairs flexibly; I will have the capital to handle the pressure of work; I will show good self-control ability. The third factor had seven questions, 21, 22, 23, 24, 25, 26, and 29. These items focused on the situation of college students’ interpersonal network in the future job search and work. Three items were also removed from this section (I will get along well with others at work; I will be able to cooperate with others at work. I will be able to handle the work conflicts I face). The fourth factor had seven questions, which were 38, 45, 46, 47,48,49, and 50. These items mainly described the ability of college students to improve their personal careers in future career development and were labeled as “career development”. Because “job search strategy” and “career development” were combined in the exploratory factor analysis, we omitted more items in this section to ensure there was no ambiguity. In this section, we deleted some items (e.g., I will be able to make a career plan based on my career goals, I will be able to accurately judge the authenticity of the job information I receive, and I will keep abreast of the development trends in my chosen field).

## Study 2

### The present study

To verify the reliability and validity of the structure and scale obtained by the exploratory factor analysis, the college students were selected again to test the subjects, and item confirmatory factor analysis, reliability analysis, and validity analysis were carried out.

### Methods

#### Subject

Referring to the commonly used method (Curran, 2016; Xu and Li, 2021), 13 participants whose answers were incomplete, and 387 valid participants remained in the end. Their average age was 20.13 ± 1.968; there were 133 male students (34.4%) and 254 female students (65.6%). Among them, 174 (45.0%) came from rural backgrounds and 213 (55.0%) came from urban backgrounds. There were 105 freshmen (27.1%), 99 sophomores (25.6%), 123 juniors (31.8%), and 60 seniors (15.5%). Among the participants, 131 (33.9%) were only child and 256 (66.1%) were non-only child; 261 (67.4%) were first-generation college students and 126 (32.6%) were non-first-generation college students in their families.

#### Tools

*Future Employability Scale* This questionnaire included four dimensions: knowledge and skills, personality quality, interpersonal network, and career development. The scale adopted the Likert five-point scale from “completely disagree” to “completely agree”. The higher the score, the stronger the future employability.

*Perceived Employability Scale* Berntson and Marklund (2007) self-perceived employability scale, which mainly reflects individuals’ measurement and judgment of their own employability, was used. The scale contains five questions such as “my skills are popular in the labor market” and scored on a Likert five-point scoring method, from “completely inconsistent” to “completely consistent”. The higher the score, the higher the individual’s self-perceived employability. The internal consistency coefficient of the scale is 0.83, and the internal consistency coefficient in this study was 0.919.

*University Commitment Scale* University commitment was measured using seven items selected or adapted from the nine organizational commitment scales identified by Tsui et al. (1997) as related to an emotional commitment (Rothwell et al., 2008). A Likert five-point scale ranging from “completely disagree” to “completely agree” was used where the higher the score, the higher the university commitment. The internal consistency coefficient of the scale was 0.83 and the internal consistency coefficient in this study was 0.931.

*Academic Satisfaction Scale* Academic satisfaction was measured using the Student’s Life Satisfaction Scale compiled by Zhang et al. (2004). College students still belong to the category of young students. The life satisfaction scale of adolescent students includes 36 items in six dimensions, including friendship, family, study, freedom, school, and environment. A Likert seven-point score was used, ranging from “completely inconsistent to completely consistent”, with higher scores representing higher satisfaction. The Academic Satisfaction Scale had six questions in the academic sub-dimension, which reflects the individual’s satisfaction with learning achievement. The internal consistency reliability of this dimension was 0.71. In the use of the time to consider the approximation of individual topics and part of the topic ambiguous to make the deletion and modification of the use of five points, the internal consistency coefficient in this study was 0.925.

#### Process

Questionnaires were distributed through the Internet. Before the questionnaires were distributed, it was explained to the participants that the survey data would be used only for scientific research and that their identities would remain confidential. After data recovery, confirmatory factor analysis was performed on the recovered questionnaires using AMOS25.0, and reliability and validity tests were performed using SPSS25.0.

## Results

### Confirmatory factor analysis

**FIGURE 1:**
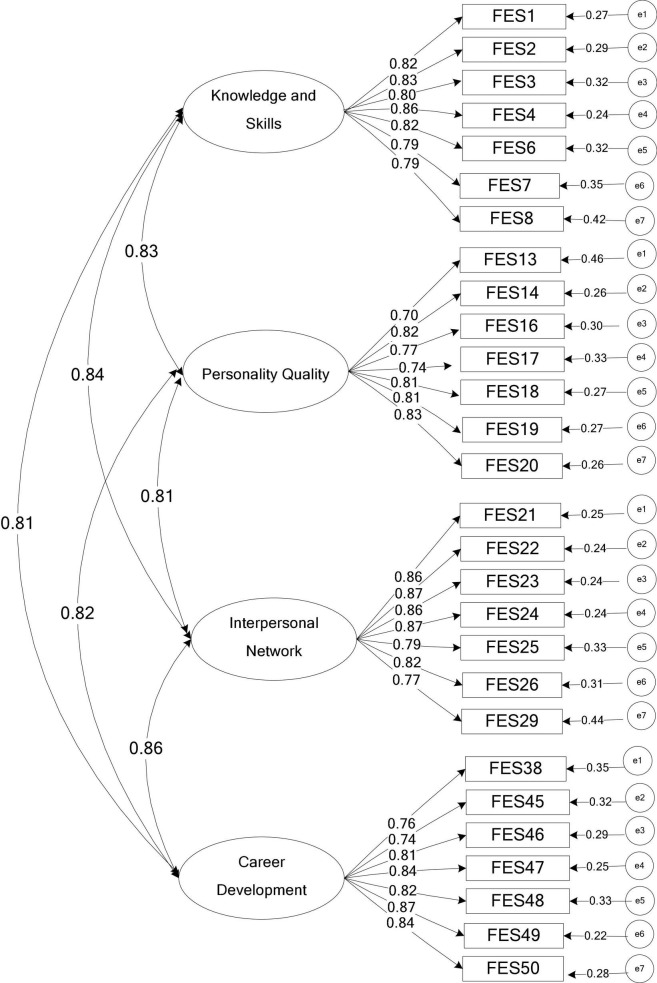
Standard solution of first-order four-factor model of college students’ future employability scale.

The structural equation model was used to compare multiple models. According to the research results of future employability, the competition models adopted in this study were the null model (M0), the first-order four-factor model (M1), and the second-order single-factor model (M2). After comparison, it was concluded that the first-order four-factor model was the best, so the first-order four-factor model (M1) was chosen (Figure 1). As evident from Table 4, χ^2^/ DF was less than 3, RMSEA was less than 0.08, and CFI and GFI were greater than 0.9. Therefore, Model 1 was finally selected in this study.

**TABLE 4:** Model fitting index.

Model	χ^2^	DF	χ^2^/DF	NFI	TLI	IFI	CFI	PCFI	RMSEA
Null model (M0)	9868.477	378	26.107						
First-order four-factor model (M1)	945.863	344	2.750	0.904	0.930	0.937	0.937	0.852	0.067
Second-order single-factor model (M2)	953.251	346	2.755	0.903	0.930	0.936	0.936	0.857	0.067

### Reliability analysis

In this study, the internal consistency coefficient and split-and-half reliability were used as reliability indexes to conduct internal analysis (*N* = 387). It can be seen from Table 5 that the total internal consistency reliability of the scale was 0.974 and that of split reliability was 0.925. The internal consistency coefficients of subscales ranged from 0.917 to 0.940. The split-half reliability of the subscales was between 0.903 and 0.928. Approximately 68 college students were selected to evaluate their retest reliability after an interval of one month. The test-retest reliability of college students’ Future Employability Scale was 0.912. The test-retest reliability for each dimension ranged between 0.798 to 0.862.

**TABLE 5:** Reliability index of college students’ future employability.

Reliability indices	Knowledge and skills	Personality quality	Interpersonal network	Career development	Future employability scale
Cronbach ɑ coefficient	0.933	0.917	0.940	0.930	0.974
Split-half reliability	0.903	0.907	0.918	0.928	0.925
Test-retest reliability	0.798	0.821	0.862	0.816	0.912

### Content validity

The results of this questionnaire were combined with the results of the research, and the psychology teachers from the university verified the accuracy of all the items and the language. They were also tasked to determine whether the problem of the students’ future employability was measured and whether the final selected items were more representative, so this scale should have higher content validity.

### Criterion correlation validity

Education and employment are two important components of an individual’s career development. Research shows that college students can predict their perceived employability, which is influenced by university commitment (Rothwell et al., 2008) and academic satisfaction (Shi, 2010; Houben et al., 2021; Presti et al., 2022a). Therefore, this study selected the perceived employability scale, university commitment scale, and academic satisfaction as the criterion. The results showed that the total score of the college students’ Future Employability Scale and the various dimensions and perceived employability scales were significantly highly related, which were relevant to the level of university commitment and academic satisfaction.

## Discussion

### Measurement perspective of future employability

This study explored the future employability of college students from the“output-based approach”. It emphasized more on the individual’s judgment of the expected future possible selves based on the current actual situation. For college students, judging their future employability from the “output-based approach” could prompt them to reflect on their assessment results and actively formulate policies to enhance their ability to achieve their career goals. Van der Heijde and Van der Heijden (2006) and Fugate and Kinicki (2008) studies on employability from the “input-based approach” focus more on the factors that influence employability. However, this study focused more on exploring individuals’ judgment on the development level of future employability from the“output-based approach”. The reason is that college students need to start from their own perspective, do a good job of self-cognition, judge their own career competitiveness, and develop specific strategies to promote their career development.

Previously, some researchers had also explored individual employability from an “output-based approach” (De Cuyper et al., 2011; Yu et al., 2014). Although the research of De Cuyper et al. (2011) and Yu et al. (2014) used the “output-based approach”, they all focused their attention on the judgment of the current situation of the individual’s own employability, which is the assessment of the real self. In addition, this study not only focused on individuals’ judgment of their own employability, but also, from the perspective of future possible self, focused on individuals’ judgment of their future possible selves, according to the current situation, and laid more emphasis on future foresight. With the arrival of the era of borderless careers, it is also crucial to actively construct future self-representation and cope with it in the dynamic career world. Therefore, from the “output-based approach”, this study actively pays attention to the future employability of college students and develops appropriate measurement tools to promote the development of research and practice.

### Structure of the future employability scale

Through item analysis and exploratory factor analysis, a 28-item college students’ Future Employability Scale was obtained. Confirmatory factor analysis confirmed that the first-order four-factor model was more accurate, which meant that the Future Employability Scale was composed of four dimensions: knowledge and skills, personality quality, interpersonal network, and career development.

In exploratory factor analysis, job search skills and career development were summed up as one dimension. This is inconsistent with the original idea that the scale of future employability would be composed of five dimensions: knowledge and skills, personality quality, interpersonal network, job-search skills, and career development. Knowledge and skills mainly describe the knowledge and skills that college students should possess for future job hunting and working processes (Yu et al., 2014), and personality quality refers to the basic quality that college students should possess in their daily work in the future (Gunawan et al., 2018). The interpersonal network defines college students’ ability to establish and use the network in the process of job search (Gunawan et al., 2018). Yu et al. (2014) also points out that “the interpersonal relationship” and “network difference” are important components of employability. Job search skills include the main strategies in the process of job search, while career development is the ability to build on individual career development in the process of career development, including the ability to get a job, choose a job, adapt to a job, maintain a job, and continue employment in the future. Li (2013) also confirms that “career development ability” is an important part of employability while job search strategy refers to a specific pre-employment strategy, which is largely an early stage of career development. Thus, it makes sense to combine job search skills with career development.

Compared with the employability scale which is based on the conservation of resources (COR) theory (Hobfoll et al., 2003) the Future Employability Scale is based on the conservation of resources (COR) theory (Hobfoll et al., 2003), the possible selves theory (Cross and Markus, 1991), and the social positioning theory (Tony, 2022), and actively pays attention to the development process of college students’ employability while focusing on the possible future self. Future employability emphasizes individuals’ perception and judgment of their own employability in the future based on the development status of employability in the current stage of career development and pays more attention to individuals’ forward-looking career development status in the future stage. In addition, compared with the future employability scale for young people compiled by Gunawan et al. (2018), which includes six dimensions (perception skills, accumulated experience, personal characteristics, interpersonal network, labor market knowledge, and education institution reputation), we aimed to measure the essential ability of college students to secure a job, maintain it, and promote career development in the future instead of including factors such as labor market demand and the reputation of educational institutions. In other words, our scale focuses more on measuring the ability and resources that an individual will possess in the future, rather than measuring the possibility of employment in the future career development process based on the demand of the labor market.

### Reliability and validity of the future employability scale

To test the reliability of the Future Employability Scale, the internal consistency reliability, retest reliability, and retest reliability were used for analysis (Table 6). From the perspective of internal consistency reliability, the reliability of the total scale was 0.974, and each dimension ranged between 0.917 and 0.940. The total split-and-half reliability was 0.925, and each dimension ranged between 0.903 and 0.928. The total retest reliability was 0.912 (an interval of one month), and each dimension was between 0.798 and 0.862. This indicates that the Future Employability Scale has good internal stability and consistency across time. The internal stability of the future employability scale is good, mainly because this study was conducted based on an extensive review of literature and matches with the survey population. The consistency of the scale across time is good because, to some extent, it indirectly reflects individuals’ judgment of their future career development status which is relatively stable in a short time interval.

**TABLE 6:** Correlation validity of college students’ future employability calibration criteria.

	Perceived employability scale	University commitment scale	Academic satisfaction scale
Future employability scale	0.838**	0.557**	0.615**
Knowledge and skills	0.725**	0.462**	0.540**
Personality quality	0.675**	0.474**	0.495**
Interpersonal network	0.808**	0.557**	0.603**
Career development	0.847**	0.538**	0.605**

**P < 0.01.

In terms of content validity, the compilation and review of scale topics strictly followed the process of scale topic development, which ensured the overall universality, representativeness, and satisfaction of the topics. Extensive solicitation of professional advice also ensured that questions accurately measured the various dimensions of future employability. In the item analysis, the three methods were integrated without deleting the topic, which indirectly reflected the good representativeness of the topic. Exploratory factor analysis found that the scale was composed of four factors. In this study, the structure of the scale was determined using the comparative competition model in confirmatory factor analysis. The results of the model comparison show that the first-order four-factor model (M1) was the most reasonable one. In terms of structural validity, exploratory factor analysis and confirmatory factor analysis cross-verified the rationality of the four dimensions of future employability (χ^2^/ DF = 2.75, NFI = 0.904, TLI = 0.930, IFI = 0.937, CFI = 0.937, PCFI = 0.852, RMSEA = 0.067), which further suggests that the structure of future employability is good.

From the perspective of criterion correlation validity, the Future Employability Scale and its dimensions are significantly positively correlated with the criterion tools. Perceived employability and employability are closely related. Individuals’ judgment of perceived employability is based on their own employability (Rothwell et al., 2008). From the results, future employability and its four dimensions are significantly correlated with perceived employability at a medium to a high level. Academic satisfaction refers to students’ overall perception of the education process compared with their preset expectations, i.e., whether an individual is satisfied with his or her current academic satisfaction status (Zhang et al., 2004). Career competencies such as knowledge, skills, and abilities that are central to career development can be influenced and developed by the individual (Akkermans et al., 2013). Presti et al. (2022b) argued that career competencies are intimately associated with employability and school-to-work transition is a fundamental career stage during which the development of career competencies can support young individuals in making a smooth transition to the labor market through active employability development activities. In addition, career competencies are intimately associated with academic satisfaction (Presti et al., 2022a). This study found that there was a medium-high positive correlation between future employability and academic satisfaction, which also confirmed Presti’s research. University commitment is derived from organizational commitment and emotional commitment. University commitment refers to the positive attitude and behavior of college students who identify with their university and are willing to make corresponding efforts. Studies show that future employability is correlated with university commitment, and verified by the study of Rothwell et al. (2008). In conclusion, the Future Employability Scale has good criterion correlation validity.

## Conclusion

In this study, the Future Employability Scale of college students was developed by reviewing literature and related tools. After item analysis, exploratory factor analysis, confirmatory factor analysis, reliability analysis, and validity analysis, the Future Employability Scale of 28 questions was finally obtained. Our research reveals that the scale is composed of four dimensions: knowledge and skills, personality quality, interpersonal network, and career development. It has good internal consistency reliability, split reliability, retest reliability, content validity, structure validity, and scale correlation validity, which can be used as an effective tool to evaluate the future employability of college students. In addition, it can promote the development of career guidance and employment guidance in universities.

## Theoretical ang practical implication

The concept of “employability” is proposed according to the university students’ competitive employment market, where the employability of the students refers to not only a certain skill or ability, or even a collection of many kinds of abilities, but one where students are also learning and developing qualities that can meet with the social demand and employment ideals. Future employability is based on the current situation of employability at the current stage from where the individual predicts and evaluates the employability that he or she will have in the future stage from a self-perspective. Based on the current stage of employability, the individual predicts and evaluates his or her own future employment, remaining employed, re-employment, and the promotion potential in future career development.

First, the concept and structure of employability and future employability have not been systematically defined in the academic community, and this study elucidates the difference and connection between them. This study clarifies the concept of employability and future employability in conjunction with existing research. Second, in recent years, with the increasing number of college graduates, the issue of employment of college students has received extensive attention from all walks of life (Rothwell et al., 2008). In this context, the Future Employability Scale provides a reliable tool for the study of future employability. In addition, in the era of borderless careers, individuals are playing an increasing role in career development. Future employability is a future-oriented concept that reflects the individual’s expected assessment of employability. It is the cognition and judgment of the possibility of self-development. The research on college students’ future employability can improve the students’ current employability. The Future Employability Scale developed in this study is reliable and will promote the development of career guidance and career consultation in colleges and universities.

## Limitations and future research direction

This study had four limitations. First, it adopted the cross-sectional research method to explore and verify the model. However, future research can also consider using the cross-sectional research method to explore the impact of college students’ future employability. Second, the scale dimensions in this study were mainly determined through a literature review. In future research, structured or semi-structured interviews can be combined to develop measurement tools. Third, the high correlation between each item and the total score may be a noteworthy problem, but confirmatory factor analysis has confirmed the reliability of the first-order four-factor model, and the model comparison also found that the first-order four-factor model is the most appropriate. Finally, the participants in this study were all Chinese college students. Whether the future employability of high school students, secondary vocational students, and foreign college students would be consistent with the future employability of Chinese college students remains to be verified further.

## Data availability statement

The datasets presented in this study can be found in online repositories. The names of the repository/repositories and accession number(s) can be found in the article/Supplementary material.

## Ethics statement

This study was approved by the Ethics Committee of Guangxi Normal University.

## Author contributions

WC: conception, collection, analysis, interpretation of data, and writing – review and editing. KS, QX, and YM: critical revision for important intellectual content. WC and KS: critical revision for theoretical framework and important intellectual content, and language expression of the manuscript. All authors approved the final version of the manuscript for submission.
