# Supplementary material for: Development and validation of Chinese college students’ future employability scale
Source: Front Psychol. 2023 Feb 23;14:1063437. doi: 10.3389/fpsyg.2023.1063437 (PMC9995856; doi:10.3389/fpsyg.2023.1063437)
Supplement: Supplementary file 1 [file Table_1.docx]

**Appendix 1. Chinese version of the Scale**

大学生未来就业能力量表（中文版）

指导语：请您基于当前阶段的实际情况，围绕您在未来就业时和转换工作时的能力做出判断。请您仔细阅读题目并在最符合的数字上打“√”。

| **题目** | **选项** | | | | |
| --- | --- | --- | --- | --- | --- |
|  | **非常不同意…………………非常同意** | | | | |
| 1. 我将具备工作所需的专业知识； | 1 | 2 | 3 | 4 | 5 |
| 2. 我将具备工作所需的专业技能； | 1 | 2 | 3 | 4 | 5 |
| 3. 我将具备工作所要求的通用技能（如计算机能力、写作能力、外语能力……）； | 1 | 2 | 3 | 4 | 5 |
| 4. 我将具备工作所需要的问题解决的能力； | 1 | 2 | 3 | 4 | 5 |
| 5. 我将具备工作所需要的创新创造能力； | 1 | 2 | 3 | 4 | 5 |
| 6. 我将具备工作所需要的组织管理能力； | 1 | 2 | 3 | 4 | 5 |
| 7. 我将有足够的实践经验用于求职； | 1 | 2 | 3 | 4 | 5 |
| 8. 我将对工作充满热情； | 1 | 2 | 3 | 4 | 5 |
| 9. 我将会积极主动的完成工作； | 1 | 2 | 3 | 4 | 5 |
| 10.我将会尽职尽责的完成工作； | 1 | 2 | 3 | 4 | 5 |
| 11.我将具备良好的职业道德； | 1 | 2 | 3 | 4 | 5 |
| 12.我将在工作中展现出良好的情绪调节能力； | 1 | 2 | 3 | 4 | 5 |
| 13.我将在工作中展现出良好的协调能力； | 1 | 2 | 3 | 4 | 5 |
| 14.我将会多方面发展自己的素质； | 1 | 2 | 3 | 4 | 5 |
| 15.我将知道如何在求职过程中建立人际网络； | 1 | 2 | 3 | 4 | 5 |
| 16.我将能建立人际网络来帮找到工作； | 1 | 2 | 3 | 4 | 5 |
| 17.我将会建立人际网络来促进工作完成； | 1 | 2 | 3 | 4 | 5 |
| 18.我将利用人际网络获取更多的职业发展路径； | 1 | 2 | 3 | 4 | 5 |
| 19.我将会在未来的工作中拓宽我的人际关系网； | 1 | 2 | 3 | 4 | 5 |
| 20.我将能够处理好复杂的人际关系； | 1 | 2 | 3 | 4 | 5 |
| 21.我将会很容易同陌生人建立关系； | 1 | 2 | 3 | 4 | 5 |
| 22.我将能找到多份与自己相匹配的工作； | 1 | 2 | 3 | 4 | 5 |
| 23.我将会从所获得的多个工作机会中做出合理选择； | 1 | 2 | 3 | 4 | 5 |
| 24.我将会在未来工作中尝试新知识新技能并运用到实践中； | 1 | 2 | 3 | 4 | 5 |
| 25.我将会具备晋升的能力； | 1 | 2 | 3 | 4 | 5 |
| 26.即使未来工作单位裁员我也能留下来； | 1 | 2 | 3 | 4 | 5 |
| 27.我将能在劳动力市场中保持竞争力； | 1 | 2 | 3 | 4 | 5 |
| 28.即使未来失去工作我也能迅速再就业。 | 1 | 2 | 3 | 4 | 5 |

注：维度一：“知识技能”包括1-7题；维度二：“个性素养”包括2-14题；维度三：“人际网络”包括15-21题；维度四：“职业发展”包括22—28题。

**Appendix 2. English version of the Scale**

**College Students' Future Employability Scale (****English version)**

**Guidance:** Based on the actual situation at the current stage, please make judgments around your ability to apply for a job and change jobs in the future. Please read the question carefully and tick "√" on the number that matches the most.

| **Item** | | **options** | | | | |
| --- | --- | --- | --- | --- | --- | --- |
|  |  | completely disagree… completely agree | | | | |
| 1.I will have the expertise required for the work； | 1 | | 2 | 3 | 4 | 5 |
| 2. I will have the professional ability required for the work； | | 1 | 2 | 3 | 4 | 5 |
| 3.I will get the general ability required by the job, such as: the ability to use a computer, the ability to write, the ability to express in a foreign language; | | 1 | 2 | 3 | 4 | 5 |
| 4. I will have the ability to solve problems at work； | | 1 | 2 | 3 | 4 | 5 |
| 5. I will show my innovation ability in my future work; | | 1 | 2 | 3 | 4 | 5 |
| 6. I will show my ability of organization and management in my future work； | | 1 | 2 | 3 | 4 | 5 |
| 7. I will have enough practical experience to apply for a job； | | 1 | 2 | 3 | 4 | 5 |
| 8. I will be enthusiastic about my future work； | | 1 | 2 | 3 | 4 | 5 |
| 9. I will take the initiative to complete the future work； | | 1 | 2 | 3 | 4 | 5 |
| 10. I will show a sense of responsibility in my future work； | | 1 | 2 | 3 | 4 | 5 |
| 11. I will demonstrate good professional ethics in my future work； | | 1 | 2 | 3 | 4 | 5 |
| 12. I will show good emotion regulation ability in my future work； | | 1 | 2 | 3 | 4 | 5 |
| 13. I will show good coordination ability in my future work； | | 1 | 2 | 3 | 4 | 5 |
| 14. I will develop my qualities in many aspects in my future work； | | 1 | 2 | 3 | 4 | 5 |
| 15. I will know how to network in the future job search process； | | 1 | 2 | 3 | 4 | 5 |
| 16. I'll be able to network to help me find jobs； | | 1 | 2 | 3 | 4 | 5 |
| 17. In the future work, I will set up a network to facilitate the smooth completion of my work； | | 1 | 2 | 3 | 4 | 5 |
| 18. I will use my network to gain more career develop paths； | | 1 | 2 | 3 | 4 | 5 |
| 19. I will expand my network in my future work； | | 1 | 2 | 3 | 4 | 5 |
| 20. I will be able to handle complex relationships； | | 1 | 2 | 3 | 4 | 5 |
| 21. I will easily form relationships with strangers； | | 1 | 2 | 3 | 4 | 5 |
| 22. I will be able to find multiple jobs that match me； | | 1 | 2 | 3 | 4 | 5 |
| 23.I will make a reasonable choice from the multiple job offers I have received; | | 1 | 2 | 3 | 4 | 5 |
| 24. I will try new knowledge and new skills in future work and apply them into practice； | | 1 | 2 | 3 | 4 | 5 |
| 25. In the future work, I will have the ability to be promoted； | | 1 | 2 | 3 | 4 | 5 |
| 26. I can stay even if my workplace is laid off in the future； | | 1 | 2 | 3 | 4 | 5 |
| 27. I will be able to remain competitive in the labor market； | | 1 | 2 | 3 | 4 | 5 |
| 28. Even if I lose my job, in the future I can quickly get a job again. | | 1 | 2 | 3 | 4 | 5 |

Note: Dimension 1: "Knowledge and Skills" includes questions 1-7; Dimension 2: "Personality quality " includes 2-14 questions; Dimension 3: "Interpersonal Network" includes 15-21 questions; Dimension 4: "Career Development" includes 22 questions - 28 questions.
